# Determining the burden of foodborne hepatitis A spread by food handlers: suggestions for a targeted vaccination?

**DOI:** 10.3389/fpubh.2025.1617004

**Published:** 2025-06-25

**Authors:** Cecilia Trucchi, Filippo Del Puente, Carolina Piccinini, Marco Roveta, Marina Sartini, Maria Luisa Cristina

**Affiliations:** ^1^Local Health Unit 3, Department of Prevention, Food Hygiene and Nutrition Service, Genoa, Italy; ^2^Department of Infectious Diseases, Galliera Hospital, Genoa, Italy; ^3^Department of Health Sciences, University of Genoa, Genoa, Italy; ^4^Operating Unit Hospital Hygiene, Galliera Hospital, Genoa, Italy

**Keywords:** hepatitis A, outbreak, food, food handlers, vaccination, public health

## Abstract

**Introduction:**

Hepatitis A virus (HAV) remains a significant foodborne pathogen, particularly when food handlers serve as the source of contamination. Its high infectivity and environmental persistence allow the virus to survive on hands, surfaces, and food, facilitating widespread transmission even from a single distribution point.

**Methods:**

This systematic review, Prospero registration number: CRD420250651930, analyzed 32 studies reporting HAV outbreaks linked to food handlers to assess whether vaccination could be an effective preventive strategy.

**Results:**

Most outbreaks occurred in North America and Europe, with index cases almost exclusively identified among food workers. Outbreak sizes varied, though the majority involved fewer than 50 cases.

**Discussion:**

Studies highlighted critical challenges, including underreporting, asymptomatic cases, and delayed interventions. Control measures largely relied on immunoglobulin administration, while vaccination was rarely implemented and showed poor adherence among food service staff. Although economic analyses were limited and sometimes inconclusive, some evidence suggested potential healthcare savings from prevention efforts. Considering HAV’s high transmissibility and the difficulty of timely outbreak detection, targeted vaccination of food handlers—especially those in high-risk settings or seasonal employment—emerges as a promising method of biological risk management in food industries. These considerations could support food industries in considering vaccination as a tool to prevent foodborne HAV transmission.

## 1 Introduction

Hepatitis A, caused by the hepatitis A virus (HAV), is the most common form of acute viral hepatitis worldwide among types A, B, C, and E (1). HAV infection is globally widespread. According to the World Health Organization (WHO), there are approximately 159 million new HAV infections each year, resulting in around 1.5 million clinical cases and 39,000 deaths (2–4).

Hepatitis A may present as isolated (sporadic) cases or occur in the form of epidemics (5). Its incidence varies greatly between countries and is closely linked to factors such as socio-demographic index, hygiene, and sanitary conditions (6). While high-income countries generally maintain good hygiene standards, these remain insufficient in many low-and middle-income regions (3, 7, 8).

In developed countries, most adults are susceptible to HAV infection. Here, outbreaks are typically driven by interpersonal transmission within high-risk groups, whereas foodborne infections are more likely to cause sporadic cases (9). However several outbreaks in these regions have been associated with contaminated food (10, 11). This shift is likely influenced by increasing international travel and global food imports, which may alter the epidemiology of hepatitis A by facilitating both outbreaks in developed countries and global transmission of the virus (12, 13).

HAV is primarily transmitted through the ingestion of food or water contaminated with feces from an infected individual, or via direct contact with an infected person (14).

Even minimal quantities—such as 1,300 infectious units per gram of food—are sufficient to cause infection. This high transmission potential is partly due to the virus’s remarkable environmental stability. HAV can remain infectious in water, soil, and on contaminated surfaces (fomites).

Its persistence is further enhanced at low temperatures, which allows it to survive for extended periods in various food matrices, including leafy greens, carrots, fennel, green onions, spinach, berries, aromatic herbs, and shellfish. For instance, HAV has been found to survive for months on frozen berries and remain infectious on surfaces depending on temperature and humidity conditions (15, 16). Furthermore, under low humidity, it can persist on foods like lettuce, bell peppers, melon, and dried tomatoes.

To inactivate HAV thermally, cooking or boiling at a minimum of 85°C (185°F) for at least one minute is required. These characteristics allow HAV to remain viable throughout the entire food chain—from production to consumption—posing a risk of fecal contamination at any stage in what is known as the “farm to fork” pathway (17).

These findings confirm that HAV can persist on food long enough to threaten consumer health. While some hygiene treatments may help reduce viral load, none have proven completely effective in eliminating the virus (18).

The virus’s resistance to acidic pH enables it to reach the intestinal tract in an infectious form; it’s incubation period range from 15 to 50 days (19).

During this time, the hepatitis A virus (HAV) is excreted in large quantities in feces, reaching concentrations up to 10^11^ genome copies per gram just before symptom onset (20). After the appearance of jaundice, viral shedding decreases rapidly as anti-HAV antibodies develop. Nonetheless, infants and young children can continue shedding the virus for up to six months post-infection (21).

Food handlers play a crucial role in preventing HAV transmission. If infected, they can transmit the virus to susceptible individuals through the food they prepare (22) and have been identified as a major source of foodborne hepatitis A outbreaks (23). A single infected food handler can transmit the virus to dozens or even hundreds of individuals during food harvesting, handling, preparation, or distribution, significantly impacting public health and healthcare costs (24–26). The primary transmission route through food handlers is direct hand-to-food contact.

Throughout the food production chain, agricultural products undergo multiple stages of handling, increasing the risk of cross-contamination by infected workers or contaminated surfaces (20). Experimental studies have shown that HAV can maintain its infectivity on hands for at least four hours (27). Simply rinsing hands with water may reduce the viral load by 10 to 100 times but is insufficient for complete removal (18). Although HAV cannot replicate outside a host, such as in food and water, its low infectious dose poses a significant risk to consumers regardless of the contamination level (28).

Despite wide variation in the number of food handlers worldwide, their role in ensuring food safety is universally essential. Nevertheless, food safety strategies have generally prioritized environmental hygiene and sanitation over direct preventive measures such as vaccination. As of now, mandatory HAV vaccination for food handlers is enforced only in a few countries, such as Germany, while in most others it remains voluntary or merely recommended.

Establishing clear epidemiological links between foodborne HAV infections and specific contamination sources remains a major challenge. This difficulty stems from the global nature of food supply chains, where ingredients can originate from distant locations, be incorporated into numerous products, and become contaminated at very low levels. As a result, outbreaks are often detected too late to trace the source effectively. The foods most commonly implicated in outbreaks include shellfish, leafy greens, and both fresh and frozen fruits—particularly berries. However, due to the possibility of cross-contamination, virtually any food can be involved (20).

Therefore, understanding HAV endemicity and identifying the main sources of infection—both human and food-related—are crucial for developing targeted prevention strategies. In line with this, a recent ECDC report on hepatitis A prevention emphasizes the importance of collaboration between public health authorities and the food safety sector to help reduce the burden of foodborne infections (29).

Other narrative reviews from recent literature have examined foodborne HAV outbreaks and the central role of food handlers in transmission, highlighting the potential benefit of immunizing food workers. Such measures could enhance food safety in compliance with HACCP (Hazard Analysis and Critical Control Points) principles while also mitigating biological risk among food industry personnel (17).

The objective of this systematic review is to assess the impact of food handlers on foodborne hepatitis A (HAV) infections, examining the crucial role they play in transmitting the virus through food.

In line with PROSPERO registration standards, this review focuses on identifying and evaluating studies that explore the role of HAV vaccination in reducing the risk of transmission via food handlers. The main outcome of the review is to establish that HAV vaccination plays a significant role in preventing foodborne HAV infections transmitted through food handlers, in light of the current epidemiology of the virus.

## 2 Materials and methods

The study protocol was registered in PROSPERO (registration number: CRD420250651930). This review was conducted and reported in accordance with the Preferred Reporting Items for Systematic Reviews and Meta-Analyses (PRISMA) guidelines, ensuring compliance with current standards for systematic review reporting. The research question was formulated using the PICO framework. The study population included individuals exposed to HAV infection, with a particular focus on food handlers. The primary outcomes assessed were the prevalence of HAV infection and its association with food handling practices.

### 2.1 Data sources and search strategy

A comprehensive literature search was performed in the PubMed/MEDLINE, Scopus, Cochrane, and Google Scholar databases from inception up to December 2025 without language restriction. The search strategy included a combination of Medical Subject Headings [MeSH] terms and keywords: (“Hepatitis A” [MeSH] OR HAV) AND (“food handlers” [MeSH] OR “food contamination”) AND (“outbreak” [MeSH] OR “infection” OR “prevalence” OR “diagnosis”). MeSH terms were applied following the nomenclature and guidelines of the National Center for Biotechnology Information (NCBI).

### 2.2 Study selection

The inclusion criteria were:

1. Studies identifying a food handler as the index case and describing subsequent infection cases related to exposure to that individual.
2. Studies explicitly linking secondary infections to food handlers, emphasizing the risk of foodborne transmission.

The exclusion criteria were:

1. Studies not directly related to HAV exposure in food handlers.
2. Studies lacking sufficient data on HAV infection, prevalence, or diagnosis.
3. Studies not meeting the PICOS criteria

Studies not meeting these criteria were excluded. No restrictions were placed on publication date or language. For further details on the search strategy, see Table 1.

**Table 1:** Search strategy adopted in the present systematic review and meta-analysis.

Search strategy	Details	
Search string	(“Foodhandlers”) AND (“outbreak”) AND (“HAV”) OR (“Hepatitis A” [MeSH]) AND (“foodhandler” [MeSH]) OR (“Hepatitis A” [MeSH]) AND (“foodhandler” [MeSH]) AND (“outbreak” [MeSH])	
Inclusion criteria	P (patients/population)	Food handlers
	I (intervention/exposure)	Contaminated food
	C (comparisons/comparators)	Not applicable
	O (outcome)	To determine the role of HAV vaccination in reducing the risk of foodborne HAV transmission via food handlers
	S [study design]	RCTs, observational studies, cohort studies, and case–control studies
Databases	PubMed/MEDLINE, Scopus, Cochrane and Google Scholar	
Exclusion criteria	Articles not relevant to research on HAV exposure in food handlers; Studies without sufficient data; Studies not aligned with PICOS criteria	
Time filter	None (from inception)	
Language filter	None (any language)	

### 2.3 Data extraction and risk of bias assessment

The articles will initially be selected by screening the abstracts. Articles deemed relevant in this first phase will be read in full and further evaluated based on inclusion/exclusion criteria, study design, population characteristics, nutritional intervention, and primary outcomes. The selection process will be carried out independently by two reviewers. Their decisions will be recorded separately and compared. Any disagreements will be resolved through consensus; if necessary, a third reviewer will be consulted. A standardized data extraction form will be used to collect information on study design, population characteristics, type and dosage of supplements, primary and secondary outcomes, and follow-up duration.

The quality and potential risk of bias of the included studies will be assessed independently by four researchers using tools tailored to study type: Observational cohort and cross-sectional studies: NIH Quality Assessment Tool for Observational Cohort and Cross-Sectional Studies. Only one study was fair quality while the others were of medium quality.

**Figure 1:**
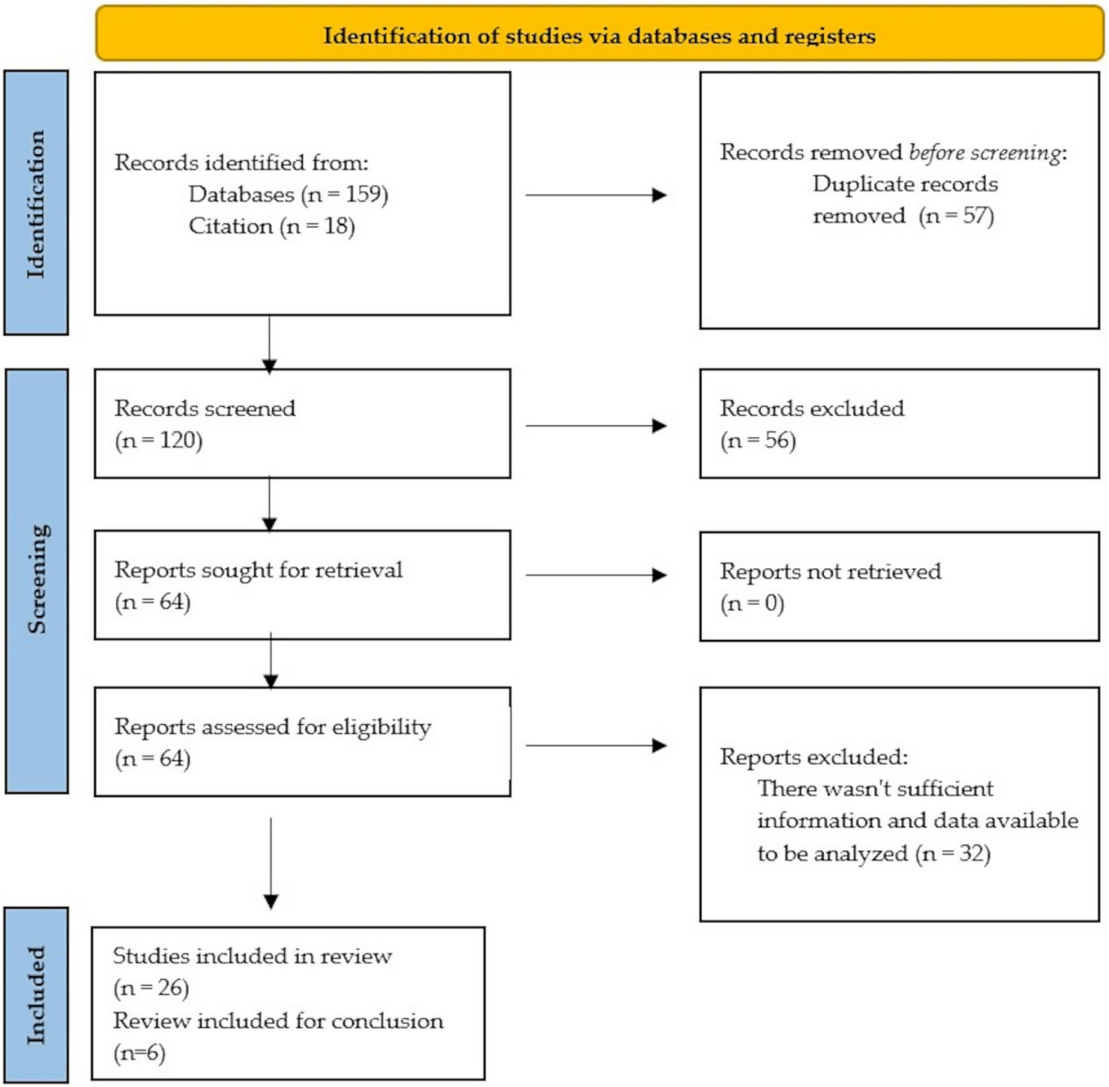
PRISMA 2020 flow diagram for new systematic reviews which included searches of databases and registers only.

In order to ensure scientific accuracy, a PRISMA flowchart was used to document the overall selection process (Figure 1).

## 3 Results

### 3.1 Study selection and characteristics

Of the 32 studies reviewed, 26 focus on specific outbreaks, while 6 are reviews. Most of the outbreak studies concentrated on single outbreaks, with only a few authors reporting consecutive outbreaks (30, 31). Among the 28 remaining studies, Tricco and Shenoy (24, 32) conducted reviews of multiple outbreaks (in Canada and India, respectively), Bidawid produced an experimental study (18), and Todd conducted a literature review (33).

Publication dates, which typically corresponded to the outbreak year or the following year, ranged from 1973 to 2020. Regarding geographical distribution, most studies were conducted in North America (15 papers) and Europe (11), with a minority in India (2) and Australia (1). Notably, studies reporting outbreaks in North America were predominantly published before 2000 (13 of 15), while all studies reporting European outbreaks were published after 2000 (11 of 11).

### 3.2 Index case characteristics

In almost all cases, the source patient was a food handler or someone who came into direct contact with food. In only one case (34), the index case was a construction worker who had nonetheless come into contact with food despite not being directly involved in the food production or distribution chain. Only three studies (35–37) reported the index case as MSM (men who have sex with men), and only Hernandez’s study reported HIV co-infection. However, it remains unclear whether other studies actually investigated these aspects of the source patient (comorbidities or sexual orientation), and therefore whether the relatively low rate of HIV co-infection or homosexuality reflects an actual absence of these factors in the “source” population or results from underreporting by the authors.

A summary of the studies reporting individual outbreaks with available data on the number of cases, country, and year is presented in Table 2.

**Table 2:** Summary of HAV individual outbreaks linked to food handlers included in the review.

Authors	Country	Year	Index case(s)	Number of cases	Investigation	Intervention
Chironna et al. (49)	Italy	2002	22-year-old local delicatessen (sandwiches)	26 cases	Case–control study, viral molecular analysis, phylogenetic relationship	N.A.
Ciesla et al. (30)	Poland	2017	34-year-old, local kindergarten, cook	32 children, 7 participants to an open-air event	Serological study, biochemical analysis	Active prophylaxis (only 2 individuals undergo despite wide availability of vaccines)
Denes et al. (31)	USA	1975	Food handler, fast food (Portland), “salad boy” (Buffalo)	22 cases (Portland) 26 cases (Buffalo)	Case–control study, restaurant inspection, index case investigation	Immune globulin to staff and patrons, surveillance of staff hygiene education
Massachusetts Department of Public Health (54)	USA	2001	Food handler (sandwiches)	21 cases	Case–control study, serological study, viral molecular analysis	Immune globulin to staff and patrons
Fortin et al. (35)	Canada	1995	23-year-old MSM cook, restaurant	6 cases	Contact tracing via temporal analysis, no case–control, serological study, and laboratory analysis.	Immune globulin to employees and sex contacts, hygiene education,
Hall et al. (39)	England	2012	Food handler, hotel	No cases	Risk assessment investigation, contact tracing, outbreak prevention response	Vaccination of staff and patients
Hanrahan et al. (40)	USA	1981	Food handler, cafeteria (sliced cold meat sandwich preparation)	37 primary cases, 73 household contacts	Case–control study, serological study for hepatitis A, food service investigation	Immune globulin to staff, reporting to health authorities, employee exclusion
Harries et al. (46)	Germany	2012	Bakery employee	83 cases	Case–control study, molecular analysis, environmental surface sampling in bakery shops, phylogenetic analysis	HAV vaccination of employees and household contacts, exclusion of infectious staff, disinfection
Hernández et al. (36)	Spain	2017	Bakery employee MSM, HIV-positive	15 cases	Questionnaires, serological study, viral nucleic extraction, phylogenetic analysis	Vaccination of employees, reinforced hygiene measures, disinfection
Honish and Bergstrom (57)	Canada	2001	Food handler, grocery store deli	N. A.	Case surveillance, contact tracing with visitor,	Recall of foods, immune globulin prophylaxis, communication campaign.
Hooper et al. (28)	USA	1974	Food handler at Dining Hall	137 cases	Case–control study, questionnaire, serological study, contact tracing.	Enhanced food safety measures, screening program for hepatitis detection, improved hygiene protocols
Kosatsky et al. (53)	USA	1982	Two MSM food handlers	3 linked outbreaks: 42 cases	Contact tracing, case–control study, serological study, sexual contact investigation	Immune globulin to contacts, enhanced hygiene measures
Kurup et al. (34)	India	2016	Construction worker who resided with hotel staff	385 cases	Case–control study, serological study, environmental investigation	Exclusion of infected food handlers, enhanced hygiene measures
Levy et al. (55)	USA	1974	Sandwich-maker at department store restaurant	107 cases	Case–control study, questionnaire, serological study, contact tracing, environmental investigation	Immune globulin prophylaxis, enhanced food safety measures, improved hand hygiene protocols
Lowry et al. (37)	USA	1986	25-year-old, MSM pantry worker	103 cases	Case–control study with temporal analysis, serological study, environmental inspection, employee investigation	Immune globulin administration, improved food safety measures, enhanced hygiene protocols
Marosevic et al. (47)	Germany	2017	Sales assistant butchers shop	12 cases	Contact tracing, stool examination, environmental investigation, molecular analysis	Post-exposure prophylaxis vaccination, contact isolation
Massoudi et al. (41)	USA	1994	Foodhandler at a catering company	91 cases	Serological study, environmental investigation, retrospective analysis	Immune globulin to employees
Meyers et al. (58)	USA	1973	Dietary employee	66 cases	Questionnaire, serological study, investigation of food preparation	Employee health monitoring, recommendation for food handlers
Nicholls et al. (48)	England	2001	47-year-old woman, catering staff	2 cases	Case identified through routine laboratory notification, contact tracing	Immune globulin prophylaxis, contact immunization at GP practice
Prato et al. (50)	Italy	2022	22-year-old food handler (grocery store)	26 cases	Case–control study, questionnaire, molecular analysis	Post exposure immunization, control measures for food handling practices
Robesyn et al. (51)	Belgium	2004	33-year-old food handler	269 cases	Case–control study, questionnaire, contact tracing, phylogenetic analysis	Enhanced surveillance and case finding, contact tracing
Rowe et al. (52)	Australia	2008	Co-owner of cafè in Melbourne	15 cases	Case series investigation, questionnaire, environmental inspection, review of staff illness registers, serological study	Media release for public awareness and case finding, post-exposure prophylaxis
Schmid et al. (42)	Austria	2007	45-year-old food handler	21 cases	Case series investigation, serological study, questionnaires, environmental investigation.	Mass vaccination campaign, enhanced hygiene measures
Schoenbaum et al. (44)	USA	1968	Baker’s assistant	74 cases	Case–control study, environmental assessment, biochemical analysis of employees	Immune globulin administration, enhanced hygiene practices
Weltman et al. (45)	USA	1994	Baker	79 cases	Case–control study	Bakery closure, immunoglobulin clinics, employee study and exclusion
Wensley et al. (43)	England	2019	Food handler in school kitchen	33 cases	Case–control study, environmental investigation, serological study, molecular analysis	Mass vaccination campaign, food service exclusion

### 3.3 Outbreak magnitude and reporting challenges

The number of direct or indirect infections and primary and secondary cases was reported by most authors, ranging from 0 (38) to 385 cases (34). In most published works, however, the number of infections was fewer than 50 (14 of the 26 studies reporting this data). It should be noted that many authors clearly identified potential underestimation of the number of exposed individuals among their studies’ limitations (31, 38–43), related to diagnostic challenges in asymptomatic or paucisymptomatic patients (44, 45) or difficulties in case tracking (36, 46).

### 3.4 Intervention strategies

Interventions primarily focused on administering immunoglobulins directed against HAV, with few cases favoring a vaccination-based approach.

Regarding vaccination, an interesting finding was that the acceptance and administration rates, when reported (30, 43, 47) were subt-optimal. Specifically, Wensley reported that the percentage of vaccinated school staff was between 56 and 60.6%, while the number of vaccinated students in primary and secondary schools was 94 and 74.5% respectively, despite a school outbreak of 33 confirmed HAV cases (43). While this may partly be attributed to the outbreak progression and not solely to anti-vaccination sentiment among staff, it is worth noting in a review context since adherence to protective measures is fundamental for containing HAV infections.

### 3.5 Immunoglobulin prophylaxis

Regarding immunoglobulin prophylaxis, this was administered in the study by Nicholls (to 486 of 725 exposed subjects) after a delay (3 weeks post-exposure), leading the author to suggest that vaccination would have been preferable (48). Fortin also reported delays between notification to health services and the start of immunoglobulin administration (35). Conversely, Hanrahan reported administering immunoglobulins relatively quickly (an average of 12.3 days after the onset of primary cases) (49). Denes reported that immunoglobulins were administered to 11,500 people across two outbreaks but noted that effectiveness was questionable due to the extended period between outbreak onset and the immunoglobulin administration campaign (31).

Delayed reporting was also highlighted by Prato (who argued that passive surveillance would have identified only a reduced number of secondary cases—16 of the 26 actually found) (50) and Lowry, who also reported issues related to MSM among the infected individuals, potentially “masking” other transmission routes (37).

In contrast, Honish reported the possibility of rapid identification and prompt immunoglobulin administration through the establishment of a telephone hotline, although the study’s limitations included difficulty in analyzing the campaign’s effectiveness (40).

### 3.6 Hygiene measures and multimodal approaches

Regarding inspection and strengthening of hygiene measures, many studies reported this system as an essential approach to pursue in combination with other control methods (28, 31, 35, 36, 39, 42) or as a standalone measure (37, 44, 45, 51, 52).

Nearly all authors employed a multimodal approach to their respective outbreaks, often focusing on a case–control methodology that enabled identification of the infection source in a relatively timely manner. In several cases (43, 47, 50, 51, 53, 54), authors utilized genomic typing to trace the outbreak progression. Massoudi and the Foodborne USA study reported that approaches based on interviews and self-assessment of hygiene practices might be ineffective due to subjectivity, leading to recall bias and data loss, thus implying the need for a multimodal approach (41, 55).

## 4 Discussion

The prevention of HAV infection through vaccination of food handlers remains an important question for public health protection. Epidemiological studies in literature are increasing in number and quality, with access to more data and information and the ability to utilize genetic sequencing techniques. Alternative but valid systems, such as computerized databases to analyze food consumption and processing, allow us to obtain valuable, important, and potentially real-time information on epidemic trends.

However, significant caveats remain in outbreak management: numerical underestimation (both in the number of outbreaks and in the number of cases within a single episode), the risk of recall bias for past events, and the time factor, which is fundamental in the epidemiological approach.

Observational studies dominate the literature and exhibit a moderate risk of bias. These studies often fail to systematically report vaccination data. Based on the GRADE methodology, these limitations translate into a *very low certainty of evidence about recommendation* for vaccination of food handlers against HAV. This rating reflects the overall quality of evidence, which is considered *very low*, and the presence of significant methodological and practical limitations that hinder a robust assessment of the intervention’s effectiveness (see Table 3).

**Table 3:** GRADE assessment.

Finding	Studies that contributed to the review finding	Study design	Risk of bias	Inconsistency	Imprecision	Indirectness	Publication bias	GRADE Level of certainty	Explanation
Vaccination of foodhandlers could reduce HAV outbreaks among the population?	All studies	Observational studies	Moderate	Not applicable	Not applicable	Serious concern	Not applicable	Very low	Foodhandler vaccination could reduce HAV outbreaks among the population, but the evidence is very uncertain

Also noteworthy is adherence to control and prevention measures, which remains difficult to evaluate and often subject to biases related to staff self-assessment, and finally, food service personnel’s willingness to undergo vaccination.

The CDC suggests a cautious approach to vaccination, strongly recommending it for personnel at risk for other reasons (staff returning from travel to endemic areas or MSM). Finally, it should be emphasized that the current social climate leads many people to find temporary or even seasonal jobs in food service, thereby exponentially expanding the pool of individuals who should undergo HAV vaccination and making monitoring virtually impossible. It is therefore crucial to implement the education about food safety among both permanent and temporary food handlers.

The public health “one-size-fits-all” approach is therefore potentially burdened with problems, as is the approach tied to profiling MSM individuals or those who have traveled from endemic countries.

From the perspective of individual food business owners, the direct economic benefits of vaccinating their employees, although not immediately apparent—especially in regions with a low incidence of HAV—could be considered both to prevent business disruptions resulting from infectious episodes and to comply with major food safety regulations.

Specifically, the European Regulation EC852/2004 (Annex 2) requires that “…No person suffering from or being a carrier of a disease likely to be transmitted through food or afflicted, for example, with diarrhea is to be permitted to handle food or enter any food-handling area in any capacity if there is any likelihood of direct or indirect contamination. Any person so affected and employed in a food business and who is likely to come into contact with food is to report immediately the illness or symptoms, and if possible, their causes, to the food business operator (56).

In conclusion, although the literature suggests that HAV vaccination can be an effective tool for preventing outbreaks, employer and employee participation in vaccination within the food industry could be encouraged through public incentives or subsidies.

Further, the improvement of surveillance and reporting of HAV cases would be invaluable in order to provide better data for assessing the risk associated with food handlers and for informing targeted prevention strategies.

## Data Availability

The original contributions presented in the study are included in the article/supplementary material, further inquiries can be directed to the corresponding author.
